# Cardioprotective and anti-hypertensive effects of *Prosopis glandulosa* in rat models of pre-diabetes

**DOI:** 10.5830/CVJA-2012-069

**Published:** 2013-03

**Authors:** B Huisamen, C George, S Genade, D Dietrich

**Affiliations:** MRC DDP, Parow, South Africa, Faculty of Health Sciences, University of Stellenbosch, Tygerberg, South Africa; Department of Biomedical Sciences, Division of Medical Physiology, Faculty of Health Sciences, University of Stellenbosch, Tygerberg, South Africa; Department of Biomedical Sciences, Division of Medical Physiology, Faculty of Health Sciences, University of Stellenbosch, Tygerberg, South Africa; Department of Biomedical Sciences, Division of Medical Physiology, Faculty of Health Sciences, University of Stellenbosch, Tygerberg, South Africa; Department of Medical Biosciences, University of Western Cape, Bellville, South Africa

**Keywords:** Prosopis glandulosa, hypertension, cardioprotection, PKB, insulin resistance

## Abstract

**Aim:**

Obesity and type 2 diabetes present with two debilitating complications, namely, hypertension and heart disease. The dried and ground pods of *Prosopis glandulosa* (commonly known as the Honey mesquite tree) which is part of the Fabaceae (or legume) family are currently marketed in South Africa as a food supplement with blood glucose-stabilising and anti-hypertensive properties. We previously determined its hypoglycaemic effects, and in the current study we determined the efficacy of *P glandulosa* as anti-hypertensive agent and its myocardial protective ability.

**Methods:**

Male Wistar rats were rendered either pre-diabetic (diet-induced obesity: DIO) or hypertensive (high-fat diet: HFD). DIO animals were treated with *P glandulosa* (100 mg/kg/day for the last eight weeks of a 16-week period) and compared to age-matched controls. Hearts were perfused *ex vivo* to determine infarct size. Biometric parameters were determined at the time of sacrifice. Cardiac-specific insulin receptor knock-out (CIRKO) mice were similarly treated with *P glandulosa* and infarct size was determined. HFD animals were treated with *P glandulosa* from the onset of the diet or from weeks 12–16, using captopril (50 mg/kg/day) as the positive control. Blood pressure was monitored weekly.

**Results:**

DIO rats and CIRKO mice: *P glandulosa* ingestion significantly reduced infarct size after ischaemia–reperfusion. Proteins of the PI-3-kinase/PKB/Akt survival pathway were affected in a manner supporting cardioprotection. HFD model: *P glandulosa* treatment both prevented and corrected the development of hypertension, which was also reflected in alleviation of water retention.

**Conclusion:**

*P glandulosa* was cardioprotective and infarct sparing as well as anti-hypertensive without affecting the body weight or the intra-peritoneal fat depots of the animals. Changes in the PI-3-kinase/PKB/Akt pathway may be causal to protection. Results indicated water retention, possibly coupled to vasoconstriction in the HFD animals, while ingestion of *P glandulosa* alleviated both. We concluded that treatment of pre-diabetes, type 2 diabetes or hypertension with *P glandulosa* poses possible beneficial health effects.

## Abstract

Obesity and type 2 diabetes present with two debilitating complications, namely, hypertension and heart disease. The dried and ground pods of *Prosopis glandulosa* (commonly known as the Honey mesquite tree) which is part of the Fabaceae (or legume) family are currently marketed as a food supplement with blood glucose stabilising and anti-hypertensive properties in South Africa. In the past, the pods of this tree were used as the primary foodstuff for the residents of the south-western regions of the North American deserts and these trees are still widely distributed across a large portion of the south-western United States.^1^ The pods are composed of 80% carbohydrate, 13% protein, 25% fibre and 3% fat, and grinding of the plant is thought to improve its use.^2^

Obesity is currently classified as a pandemic and is recognised as the leading cause in the development of the metabolic syndrome. The metabolic syndrome is described as a cluster of pathophysiology outlined by the National Cholesterol Education Program’s Adult Treatment Panel III (NCEP: ATP III) and the European Group for the Study of Insulin Resistance, to include insulin resistance or glucose intolerance (pre-diabetes), type 2 diabetes, hypertension and atherogenic dyslipidaemia.^3^,^4^ In addition, all of these factors can be considered independent risk factors for the development of cardiovascular disease.^3^

According to the World Health Organisation (WHO), non-communicable diseases such as heart disease, stroke, cancer, chronic respiratory diseases and diabetes are currently (updated June 2011) the leading causes of mortality in the world.^5^ This invisible epidemic is an under-appreciated cause of poverty and hinders economic development in many countries. The burden is growing and the number of people, families and communities afflicted is increasing.

The time-line for development of overt type 2 diabetes is described as developing over many years. The cardiovascular consequences of this so-called ‘ticking clock’ hypothesis, starting from obesity and culminating in type 2 diabetes, is present from the early pre-diabetic stages.^6^

In view of the scarcity and cost of modern oral hypoglycaemic agents, plant-based therapies for the treatment of diabetes are gaining considerable prominence.^7^ According to these authors more than 400 plant species have been described as having hypoglycaemic activity. However, not all of these substances have been researched scientifically to validate their efficacy.

We have researched a product from one such plant species, consisting solely of the dried and ground pods of the plant *P glandulosa*, for hypoglycaemic properties.^8^ In addition, potent anti-infective and anti-parasitic compounds have also been isolated from this plant.^9^

In view of the hypoglycaemic effects of *P glandulosa*, as well as its ability to partially restore the function of pancreatic tissue and increase cardiomyocyte insulin sensitivity,^7^ we set out to determine the cardiovascular effects of treatment, using a well-characterised rat model of obesity and pre-diabetes with known cardiovascular insufficiency and endothelial dysfunction.^10^,^11^ In addition, using a rat model of high-fat feeding known to develop hypertension,^12^ we determined whether *P glandulosa* had any effects on the development of high blood pressure.

## Models

## 1. Diet-induced obesity (DIO)

As described previously,^10^ Wistar rats (180–200 g) were randomly divided into a control and diet group. The DIO group was fed a diet of normal rat chow supplemented with sucrose and condensed milk for a basic period of eight weeks. From weeks eight to 16 the rats were treated with *P glandulosa* (100 mg/kg/day) set in jelly/gelatine blocks and given to each one individually according to the weight of the animal.^8^ This was done to ensure absolute compliance and dose control. The dose of *P glandulosa* was calculated as previously described.^8^

The diet to induce pre-diabetes in the animals was based on hyperphagia.^13^ Animals were anaesthetised with sodium pentobarbital (160 mg/kg, intra-peritoneally) before experimentation. At the time of sacrifice, their body weight and the weight of the intra-peritoneal fat were noted and trunk blood was collected for biochemical analyses. For Western blot analyses, the hearts were removed, immediately snap-frozen in liquid nitrogen and stored at –80°C.

## 2. High-fat diet (HFD)

To induce high blood pressure, the rats were fed a diet containing the following per kg of food: cooking fat 400 g, fructose 100 g, casein 100 g, cholesterol 10 g, and rat chow pellets 390 g. Blood pressure was monitored on a weekly basis over 16 weeks. Treatment with *P glandulosa* (100 mg/kg/day) given in jelly blocks was either started at the onset of the diet to study the effect on prevention of the development of hypertension, or after a period of 12 weeks of the HF diet to study its anti-hypertensive effects. Rats treated with captopril (50 mg/kg/day) from the onset of the diet were included as a positive control. All animals were also placed individually in metabolic cages in order to collect urine samples.

## 3. CIRKO mice

A mouse model of animals with a cardiac conditional ablation of the insulin receptor was used in conjunction with their C57Bl6 littermates.^14^ Mice were fed normal chow and treated with *P glandulosa* at a similar dose to that of the rats for a period of eight weeks before experimentation.

## Methods

Animals had free access to food and water and were kept on a 12-hour day/night cycle in the Central Research Facility of the Faculty of Health Sciences of the University of Stellenbosch. The study conformed to the revised South African National Standard for the Care and Use of Animals for Scientific Purposes (South African Bureau of Standards, SANS 10386, 2008) and was registered with the Committee for the use of animals in research of the University of Stellenbosch – numbers P05/11/013 and P07/11/020.

The *P glandulosa* plant material was originally obtained from naturally growing plants. The material was handled according to a patented and standardised procedure^8^ and pre-packed in capsules for human consumption, which we emptied and weighed. The voucher specimen was reported previously.^8^

Plasma glucose levels were determined in the fasting state. Blood was obtained via a tail prick and glucose levels were determined using a conventional glucometer (Cipla MedPro). Plasma was stored at –80°C in a Snijders Scientific Ultracool (Tilburg, the Netherlands) and insulin levels were determined using a coat-a-count assay (Diagnostic Products).

Intra-peritoneal glucose tolerance curves (IPGTTs) were generated in the animals after an 18-hour fast. Animals were injected intra-peritoneally with 1 g/kg of a 50% sucrose solution and blood glucose levels were monitored over a 120-min period.

After removal, the hearts were arrested in ice-cold Krebs Henseleit (KH) medium (in mM: NaCl 119, NaHCO_3_ 25, KCl 4.75, KH_2_PO_4_ 1.2, MgSO_4_.7H_2_O 0.6, Na_2_SO_4_ 0.6, CaCl_2_.2H_2_O 1.25, glucose 10) and immediately (within 30 sec) mounted onto the aortic cannula of a perfusion rig. The pulmonary vein was connected to a second cannula in order to perform perfusions in the working-heart mode with a preload of 15 cm H_2_O and an afterload of 100 cm H2O, as described previously.^15^ The perfusion medium was continuously gassed with 95% O_2_ /5% CO_2_. Hearts were fitted with a temperature probe and the temperature was kept constant at 36.5–37°C.

After a stabilisation period of 30 min, rat hearts were subjected to 35-min regional ischaemia by coronary artery ligation, followed by reperfusion for one hour, as described previously.^15^ Infarct size was determined according to a well-established protocol,^15^ followed by planimetry, and expressed as a percentage of the area at risk. Planimetry was performed blind by a third party.

Mouse hearts were perfused retrogradely, meaning via the aorta without a connection to the pulmonary vein. After the 30-min stabilisation period, the hearts were subjected to 20-min normothermic ischaemic cardiac arrest (NICA) by stopping all perfusion. This was followed by one hour of reperfusion, after which the infarct development through the whole heart was determined as described above.

To measure blood pressure, rats were placed in restraining holders with a dark nose cone to calm them. The restrainers were placed on a heating pad (32 ± 2°C) to warm the rat and maintain blood flow to the tail. Animals were placed in the restrainers for at least five minutes before monitoring the blood pressure using a computerised tail-cuff blood pressure monitor (Kent Scientific Corporation, Connecticut, USA). Prior to commencement of the experiment, rats were subjected to the above procedure daily for at least a week to train the animals for the procedure and to avoid stress in the rats during experimental determinations.

Animals were placed individually in metabolic cages and the volume of urine was determined over a period of 24 hours.

## Western blotting

Frozen tissues were pulverised with a liquid nitrogen pre-cooled mortar and pestle and then extracted in lysis buffer containing in mM: Tris-HCl 20 (pH 7.5), EGTA 1, EDTA 1, NaCl 150, Na_2_VO_3_ 1, beta-glycerophosphate 1, sodium-pyrophosphate 2.5, PMSF 0.3, Triton X-100 1% (v/v) plus 10 μg/ml leupeptin and aprotinin, respectively, using a Polytron PT10 homogeniser, 2 × 4 sec, at setting 4. Lysates were cleared from particulate matter by centrifuging for 15 min at 14 000 rpm in a microfuge (Eppendorf Mini-spin plus, Hamburg, Germany) and the protein content was determined by the method of Bradford.^16^ Samples were diluted in Laemmli sample buffer, boiled for 5 min and stored at –80°C.

Equal amounts of cytosolic proteins were separated on a SDS poly-acrylamide gel and electro-transferred to ImmobilonTM-P PVDF membranes. Transfer and equal loading of proteins was determined with Ponceau red reversible stain. The membranes were blocked for two hours in Tris-buffered saline (TBS) containing 0.1% Tween-20 and 5% non-fat milk powder and incubated overnight in the primary antibodies (diluted in TBS–Tween according to the manufacturer’s instructions). The following antibodies from cell signalling were used: insulin receptor beta-subunit, phospho-PI3K P85 (Tyr458), total and phospho-PTEN (Ser380/Thr382/383), total and phospho-PKB/Akt (Ser473), Glut 1 and Glut 4

Blots were stripped using a 5-min incubation in 2% NaOH after washing in distilled water and re-probed with a beta-tubulin antibody to confirm equal loading. Bands were visualised using the ECL detection system and quantified by laser-scanning densitometry with suitable software (Silk Scientific Inc, USA). For comparison purposes, total pixels of bands were expressed as a ratio of the mean of the controls on the same blot.

## Statistical analyses

Data are presented as mean ± SEM and were analysed using either a one-way or two-way ANOVA followed by a Bonferroni *post-hoc* test for differences between groups. The blood pressure effects were analysed using a repeated-measures two-way ANOVA. Statistical significance was set at *p* < 0.05.

## Results

After the 16-week diet animals from model 1 (DIO) presented with significantly increased body- and intra-peritoneal fat weight (Table 1). As summarised in Table 1, these animals had significantly elevated blood glucose and insulin levels, leading to an increased homeostatic model assessment of insulin resistance index (HOMA-IR), indicative of whole-body insulin resistance.

**Table 1:** Biometric Data – Model 1: DIO

	*Control*	*Control + P glandulosa*	*DIO*	*DIO + P glandulosa*
Weight	433.7 ± 9.3	438.6 ± 9.3	507.7 ± 22.9***	534.3 ± 11.7***
Intra-peritoneal fat	18 ± 2.7	11 ± 1.8	28.0 ± 1.74***	34 ± 1.4***
Blood glucose (mmol/l)	5.42 ± 0.17	5.4 ± 0.18	6.4 ± 0.17*	5.6 ± 0.19
Serum insulin (μU/ml)	17.12 ± 0.8	14.07 ± 1.50	34.33 ± 9.06*	35.93 ± 10.21*
HOMA-IR	4.73 ± 0.71	3.40 ± 0.40	8.96 ± 2.65*	7.88 ± 3.30*

*p < 0.05 vs the respective control; ***p < 0.001 vs the respective control. Analysis by two-way ANOVA, *n* = 6 per group.

**Fig. 1.**
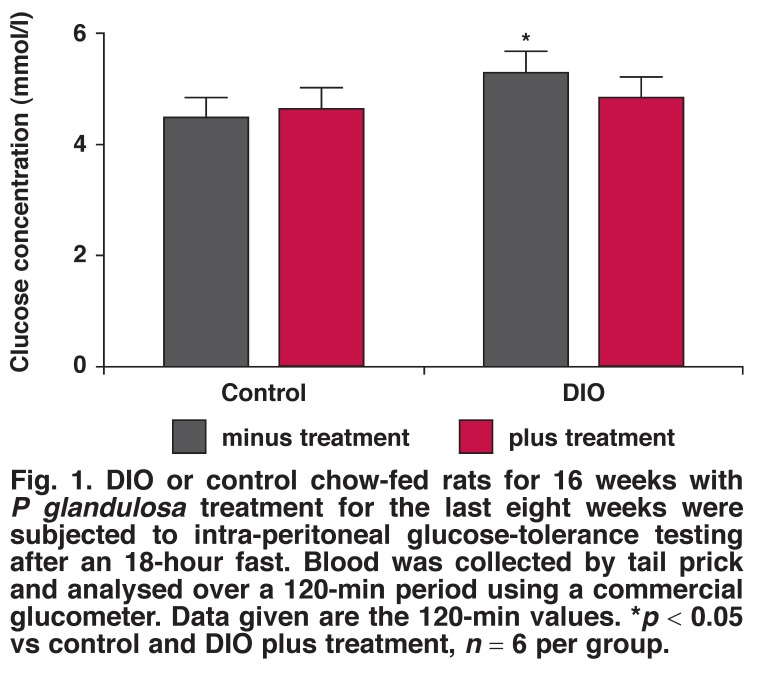
DIO or control chow-fed rats for 16 weeks with *P glandulosa* treatment for the last eight weeks were subjected to intra-peritoneal glucose-tolerance testing after an 18-hour fast. Blood was collected by tail prick and analysed over a 120-min period using a commercial glucometer. Data given are the 120-min values. *p < 0.05 vs control and DIO plus treatment, *n* = 6 per group.

In neither control nor DIO animals did the treatment with *P glandulosa* have any effect on the body weight or the intraperitoneal fat weight of the animals. After treatment of the DIO animals with *P glandulosa*, the blood glucose levels were no longer significantly elevated compared to the treated controls but the HOMA-IR was still significantly higher. However, as shown in Fig. 1, the two-hour blood glucose values after intraperitoneal glucose tolerance analyses were significantly lower in the treated DIO animals, underscoring a slight effect on blood glucose handling, as previously reported.^8^

## Infarct size

After 16 weeks of the obesity-inducing diet, the *ex vivo* perfused hearts of the DIO animals presented with significantly larger infarct sizes, calculated as percentage of the area at risk, than the hearts from the control animals (DIO 49.48 ± 3.25 vs control 40.62 ± 2.21%, *p* < 0.05, *n* = 17 per group). The area at risk did not differ between the groups and averaged 54.13 ± 2.21%.

**Fig. 2.**
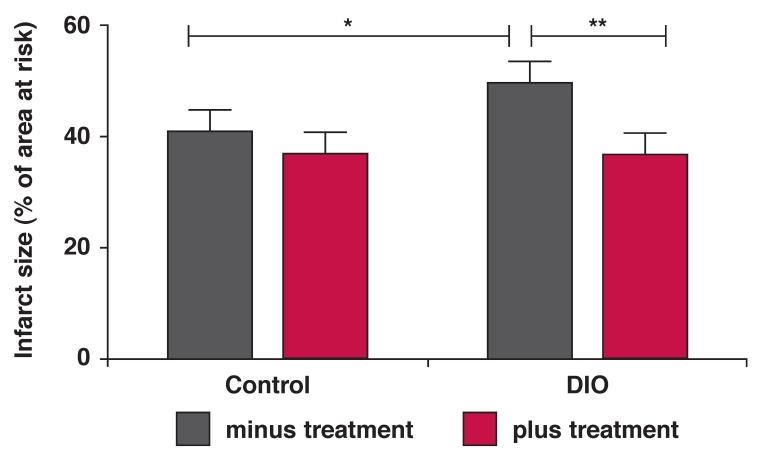
After the 16-week diet plus *P glandulosa* treatment, isolated hearts from DIO rats were perfused *ex vivo* in the working-heart mode. They were subjected to regional ischaemia as described in Methods. Infarct size was determined as a percentage of the area at risk of infarction. *p < 0.05, **p < 0.01, *n* = 15–17 per group.

An eight-week treatment regime with *P glandulosa* in conjunction with the diet significantly improved the ability of the hearts to withstand a period of ischaemia, and smaller infarcts developed. There was no significant effect in the hearts from control rats (Fig. 2). Two-way ANOVA indicated a significant effect of the treatment on infarct size (*p* < 0.01).

**Fig. 3.**
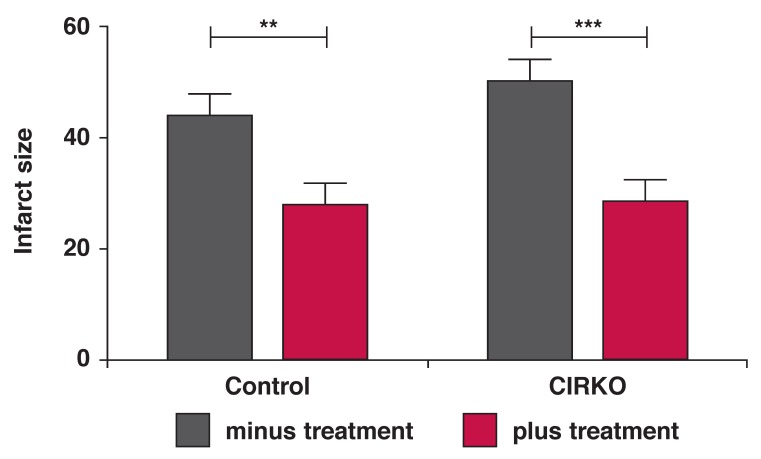
After the eight weeks of treatment, hearts were removed from the CIRKO mice and perfused ex vivo in the Langendorff mode and subjected to NICA as described in Methods. Infarct size was determined throughout the whole heart and expressed as a percentage of the total surface. **p < 0.01, ***p < 0.001, *n* = 9 per group.

To confirm these results and rule out any effect of insulin levels on the cardioprotective role of *P glandulosa*, we used a mouse model with a conditional ablation of the insulin receptor in cardiomyocytes.^14^ Subjecting these animals and their normal C57Bl6 littermates to *ex vivo* perfusion and NICA, followed by reperfusion, we found that the hearts of both control and CIRKO mice were protected by the *P glandulosa* treatment. This was demonstrated by the significantly smaller infarct size observed (Fig. 3). The effect of this treatment was highly significant (*p* < 0.001, *n* = 9 per group) as indicated by two-way ANOVA.

## Analyses of proteins forming part of the insulinsignalling cascade

Protection against myocardial damage induced by ischaemia–reperfusion and culminating in the formation of an infarct has been ascribed, among others, to the activity of the phosphatidylinositol-3-kinase (PI-3K) pathway. In view of the previously reported improvements in insulin sensitivity of cardiomyocytes, induced by *P glandulosa* treatment,^8^ we systematically analysed the proteins involved in this signalling cascade.

**Fig. 4.**
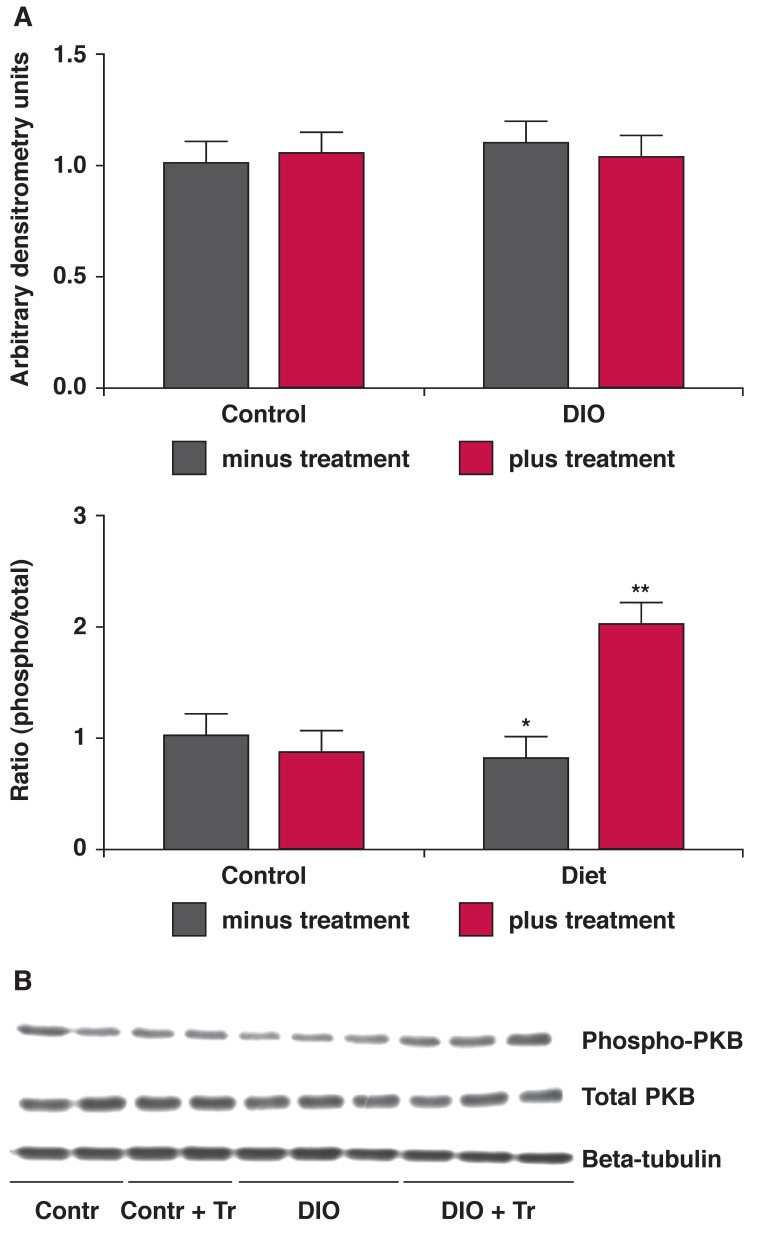
Hearts from the treated and untreated DIO animals were removed without any intervention and stored in liquid nitrogen. Tissue lysates were prepared and Western blotting was performed as described in Methods. A: bar charts of the expression of PKB protein as well as the ratio of phosphorylated vs total protein. *p < 0.05 vs control; **p < 0.01 vs untreated DIO, *n* = 6 individual hearts analysed per group. B is a representative blot depicting these proteins and beta-tubulin, used as an indicator of equal loading.

As summarised in Table 2 and shown in Fig. 4, hearts from the DIO animals presented with a significantly lower phosphorylated:total ratio of the central protein in this cascade, protein kinase B or Akt. This ratio was significantly improved by treatment. In addition, the expression of the p85 regulatory subunit of the PI-3K enzyme was significantly lower in hearts from the DIO animals, whereas this was not the case after treatment.

**Table 2:** Summary Of The Western Blot Analyses Of The Proteins Involved In The Insulin Signal Transduction Pathway With Arrows Indicating The Effect Induced By The Diet Alone Or The Diet In Combination With *P Glandulosa* Treatment. Hearts Were Freeze-Clamped In The Basal State Without Any Interventions

*Protein*	*Effect of diet*	*Effect of treatment*
Glut 1	↔	↔
Glut 4	↔	↔
IR-beta	↔	↔
PKB/Akt	P/T ↓	P/T ↑
p85	↓	↔
PTEN	↔	T ↓ P/T ↑

P = phosphorylated protein, T = total protein; P/T = the ratio of phosphorylated to total protein, *n* = 6 individual hearts per group.

**Fig. 5.**
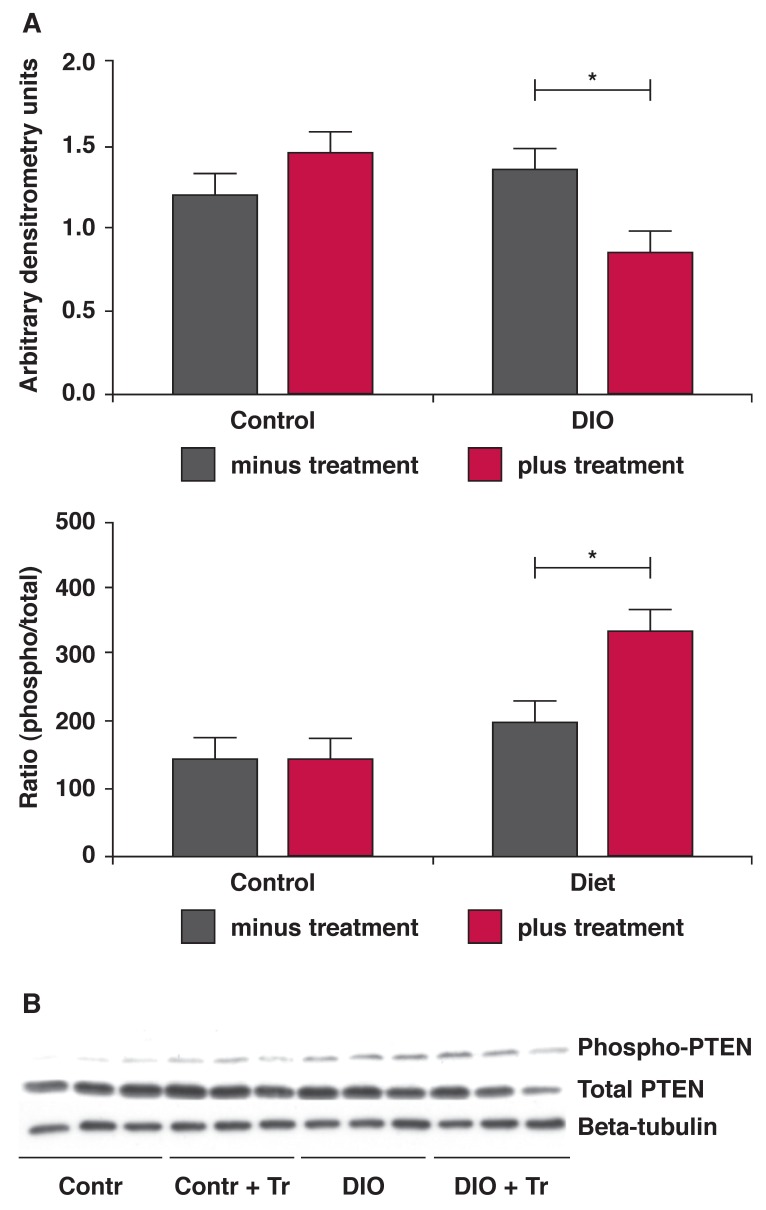
Hearts from the treated and untreated DIO animals were removed without any intervention and stored in liquid nitrogen. Tissue lysates were prepared and Western blotting was performed as described in Methods. A: bar charts of the expression of the PTEN protein as well as the ratio of phosphorylated vs total protein. *p < 0.05, *n* = 6 individual hearts analysed per group. B is a representative blot depicting these proteins and beta-tubulin, used as an indicator of equal loading.

Treatment also resulted in a lower expression of the phosphatase and tensin homologue deleted on chromosome 10 (PTEN) with a higher state of phosphorylation of this enzyme (Fig. 5). Phosphorylation of PTEN further inactivates this enzyme, responsible also for the dephosphorylation of PKB/ Akt.^17^,^18^

## Anti-hypertensive effects

As the DIO diet does not cause high blood pressure, we used a modification of a high-fat diet to induce hypertension in the animals.^12^ As can be seen in Fig. 5, these animals developed a significant elevation of their blood pressure within four weeks (HFD 135.88 ± 2.0 vs control 125.85 ± 1.9 mmHg, *p* < 0.05, *n* = 8 per group).

We either pre-treated the animals with *P glandulosa*, starting at the onset of the diet, or we allowed the animals to become severely hypertensive (12 weeks) and then started the treatment. We included a group of animals treated with the angiotensin converting enzyme (ACE) inhibitor captopril from the onset of the diet, as a positive control in this study.

**Fig. 6.**
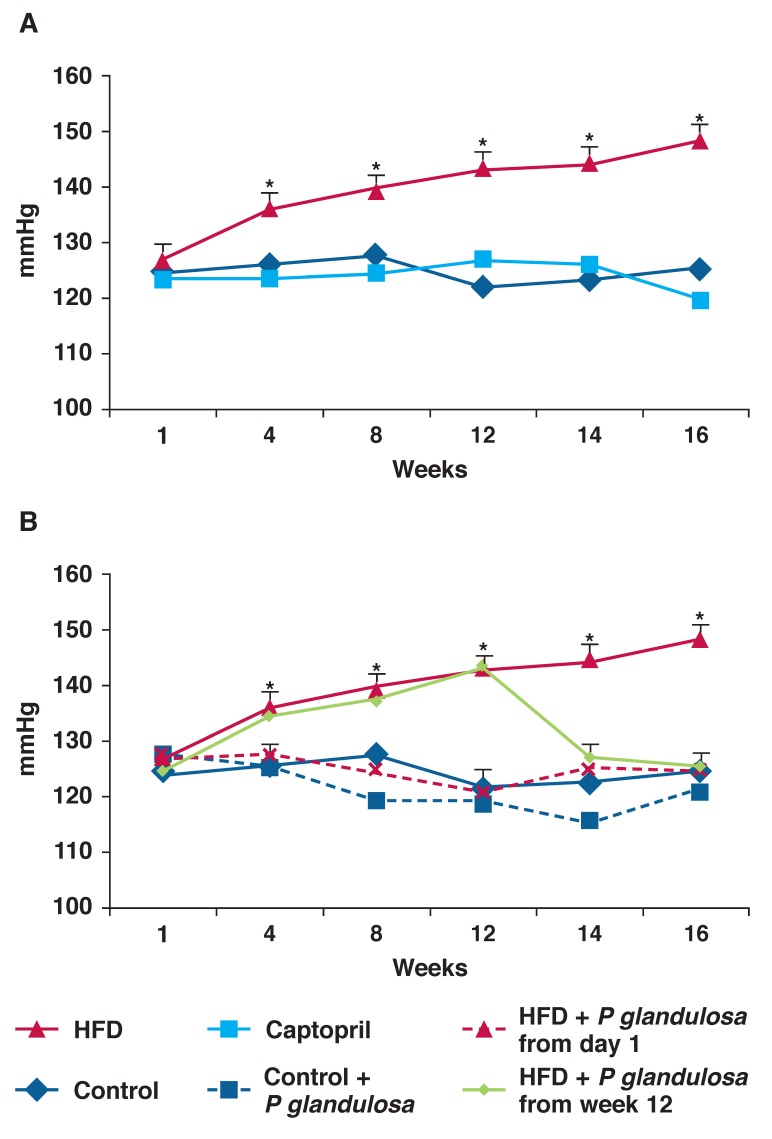
Rats were fed a high-fat diet for 16 weeks and blood pressure was monitored on a weekly basis as described in Methods. *p < 0.001 vs control and captopril, *n* = 9 per group. A: HFD vs captopril, B: HFD vs P glandulosa treatment.

As can be seen in Fig. 6A, captopril prevented the development of hypertension in the animals. Similarly, *P glandulosa* treatment prevented the development of high blood pressure in these animals when given in conjunction with the high-fat diet. *P glandulosa* treatment did not significantly affect the animals on the control diet (Fig. 6B). In addition, treatment of already hypertensive animals (week 12) with *P glandulosa* normalised their blood pressure within two weeks.

## Effects on urine production

Measuring the urine output of the animals by keeping them separately in metabolic cages showed that after the 12-week treatment period, the urine output of animals on the control diet was 17.37 ± 0.8 ml while those on the high-fat diet had a significantly lower urine output of 9.8 ± 0.55 ml (*p* < 0.001, *n* = 9 per group).

**Fig. 7.**
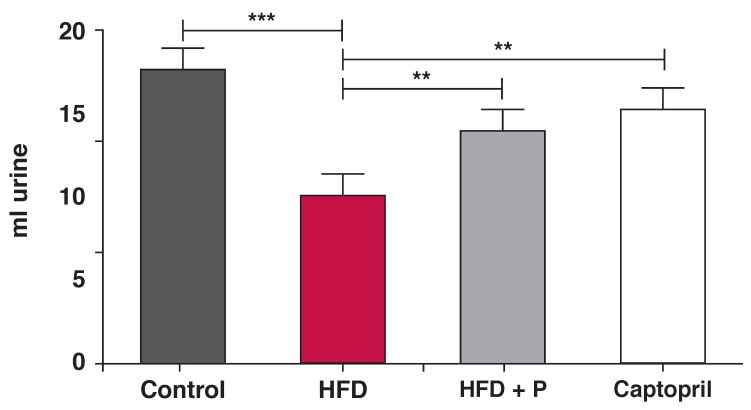
Rats on the high-fat diet were individually placed in metabolic cages for the collection of urine over a 24-hour period. Data were collected at 12 weeks after the diet was started. **p < 0.01, ***p < 0.001, *n* = 9 per group.

Captopril treatment elevated the urine output to 15 ± 0.9 ml. Treatment with *P glandulosa* also elevated urine output to 13.68 ± 0.80 ml (*p* < 0.01, *n* = 9 per group) (Fig. 7).

## Discussion

Currently, the world is suffering from a silent epidemic starting with obesity and culminating in type 2 diabetes.^3^ Two of the most debilitating complications of obesity, especially centrally located obesity, responsible for the high morbidity and mortality associated with such patients are hypertension and heart disease.^19^,^20^ In view of the need for effective medication to supplement lifestyle changes to control these disease states, utilisation of plant-based therapies are currently strongly advocated.^7^,^21^ Such therapies offer potentially cost-effective management but need scientific validation of their effects.

Previous studies from our laboratory demonstrated that the dried and ground pods of the *P glandulosa* tree have a potential benefit in the management of both type 1 and type 2 diabetes.^8^ In view of the insulin-sensitising effects on isolated cardiomyocytes from rats treated with *P glandulosa*, we aimed to determine whether this product has any cardioprotective or anti-hypertensive effects.

In this study, we used three different animal models. The first was a model of pre-diabetes (DIO), as also indicated by the biometric data presented in Table 1. These animals were fed an obesity-inducing diet containing only 16% fat.^11^,^13^ DIO animals become insulin resistant but not diabetic, as the blood glucose levels never rose above ~ 6.5 mmol/l. This was however significantly higher than the levels found in the control, chowfed animals. In order to keep the blood glucose levels low, the animals presented with high plasma insulin concentrations.

Although *P glandulosa* treatment did not significantly alter these parameters, the clinically important two-hour blood glucose values after a glucose tolerance test were significantly higher in the DIO animals and were effectively lowered by the treatment (Fig. 1). This underscores the slight effect on blood glucose handling previously reported.^8^

Determination of infarct size in ex vivo perfused rat hearts as a measure of myocardial damage incurred by ischaemia followed by reperfusion, is taken as the gold standard to prove cardioprotection.^15^ We previously showed that hearts from the DIO rats developed larger infarct sizes when subjected to regional ischaemia followed by reperfusion.^10^

After eight weeks of treatment of DIO rats or CIRKO mice with *P glandulosa*, it was clearly demonstrated that there was an infarct-sparing effect elicited by ingestion of this plant material (Figs 2, 3). As the CIRKO mice do not possess a myocardial insulin receptor, the protection found in these animals confirmed the results obtained in the rat model and underscores that protection does not occur via the insulin-secretory effects of *P glandulosa*, as previously reported.^8^

One of the best-described and researched mechanisms of protection of the heart against ischaemia–reperfusion injury and infarction is activation of the PI-3K, PKB/Akt pathway, normally activated by various extracellular substances.^22^-^24^ Activation of this pathway has several anti-apoptotic effects, leading to limitation of the development of an infarct after ischaemia.

In addition, activation of PKB/Akt is a pre-requisite for glucose uptake by the heart.^25^ Myocardial glucose is taken up via the two transporters Glut 1 and Glut 4. An improved ability to import and utilise glucose is cardioprotective when the heart is subjected to the absence of oxygen, as induced by ischaemia. The heart then uses the energy generated by glycolysis to protect itself.

Measurement of the expression of both Glut 1 and Glut 4 showed no differences between hearts from control and DIO rats. However, the lower ratio of phosphorylated to total protein of PKB/Akt found in hearts from the DIO animals may have been detrimental during an ischaemic incident. In addition, there was lower expression of the p85 subunit of PI-3K documented in these hearts, which may have exacerbated this effect.

Both of these detrimental changes were improved by *P glandulosa* treatment. The changes documented in the phosphatase PTEN will further the positive effects found in both PI-3K and PKB/Akt as the lower expression and elevated phosphorylation of this enzyme will elevate the activity of PKB/Akt when the latter is stimulated.^18^ PTEN normally inactivates PKB/Akt.^17^ These changes may play a central role in the protection that *P glandulosa* treatment confers on the heart.

The second rat model was aimed at specifically inducing the development of hypertension. A modification of a high-fat diet was used (HFD).^12^ These animals, in contrast to the DIO animals, developed severe hypertension within a four-week period, as shown in Fig. 6A and B. Not only was *P glandulosa* treatment able to prevent the development of hypertension when given in conjunction with the high-fat diet, but it normalised elevated blood pressure within two weeks.

The hormonal effects associated with a high-fat diet in rats, namely elevated vasopressin as well as activation of the renin–angiotensin system, leading to elevated aldosterone levels may both be involved in the development of hypertension in these animals.^26^-^28^ Vasopressin, the anti-diuretic hormone leads to water retention and therefore the development of high blood pressure. In addition, it is associated with vasoconstriction.^28^ Similar effects can be expected from elevated sympathetic activity, leading to elevated aldosterone levels. Measuring the 24-hour urine output of the HFD animals underscored this, as the HFD animals had a significantly lower urinary output than the controls.

According to Lee and Blaufox,^29^ a volume of 16–17 ml urine can be expected from normal animals in the weight range of our experimental rats (control 258.49 ± 15.03 vs HFD 327 ± 12.90 g, *p* < 0.05, *n* = 14 per group) while a high-fat diet will result in concentration of this volume, indicating water retention. It can also be speculated that, in parallel with the latter effect, there will be vasoconstriction, contributing to the observed hypertension.

The treatment with *P glandulosa* was able to alleviate this, thereby adding to the myocardial protection observed. To highlight this argument, in the present study both the ACE inhibitor and *P glandulosa* treatment significantly improved urinary flow of the animals, in conjunction with lowering the blood pressure. Although this was not measured in the current study, we speculate that vasopressin production and aldosterone levels were elevated in the HFD rats. *P glandulosa* treatment may affect the levels of either of these hormones, or it may provide a different, hitherto unrecognised mechanism of lowering blood pressure in the animals.

## Conclusion

The present study has confirmed our previous results that the dried and ground pods of the *P glandulosa* tree have anti-hyperglycaemic effects. In addition we have conclusively shown that this treatment was cardioprotective, as determined by the infarct-sparing effects, and anti-hypertensive without affecting the body weight or the intra-peritoneal fat depots of the animals. The results indicated that key proteins involved in the cardioprotective PI-3-kinase/PKB/Akt pathway were affected in a manner that may be causal to this protection.

With regard to the anti-hypertensive effects, the results indicated water retention, possibly coupled with vasoconstriction in the HFD animals, while ingestion of P glandulosa alleviated both water retention and hypertension. Treatment of pre-diabetes, type 2 diabetes or hypertension with P glandulosa therefore poses potentially beneficial health effects besides its anti-hyperglycaemic effects.
